# Parental weight changes as key predictors of child weight changes

**DOI:** 10.1186/s12889-015-2005-x

**Published:** 2015-07-12

**Authors:** Helen Andriani, Chu-Yung Liao, Hsien-Wen Kuo

**Affiliations:** International Health Program, National Yang-Ming University, Taipei, Taiwan; Department of Early Childhood Educare, College of Health, Chung Chou University of Science and Technology, Changhua, Taiwan; Institute of Environmental and Occupational Health Sciences, National Yang-Ming University, Taipei, Taiwan; School of Public Health, National Defense Medical Center, Taipei, Taiwan

## Abstract

**Background:**

Parents are the key agents of behavioural changes in their children. This fact is as an important aspect of obesity treatment and prevention. The present study aims to evaluate the influence of parents who have gained or lost weight on their children’s weights and to examine parental and child patterns of weight changes from a baseline over a 14-year duration.

**Methods:**

We performed a secondary analysis on the Indonesia Family Life Survey (IFLS), an ongoing national prospective longitudinal cohort study in Indonesia. Height and weight measurements, information regarding parental education, maternal employment, household income, and residence were collected from children under five years old (n = 3,147) and their parents in 1993. Data were taken from the same individuals at different points in time, in 1997, 2000, and 2007.

**Results:**

During each transition, the children of parents who gained weight had a significantly weights than did children of parents who lost weight. A mother’s positive weight change increased the chance of her pre-schooler’s or school-aged child’s positive weight change. However we found no such association between a father’s positive weight change and his child’s positive weight change.

**Conclusions:**

Parental weight change is an independent predictor of child weight change. Positive weight change in the mother had a more dominant influence than did the father’s positive weight change. Future family-based obesity prevention and treatment programs should consider how best to include and engage mothers as a catalyst for the reduction of obesity-related risk factors in the long term.

## Background

The increasing prevalence of overweight and obesity in children in both developed and developing countries [1] has spawned research investigating modifiable predictors of excessive weight gain in childhood. Excess weight in childhood is linked to many adverse health consequences, a wide range of serious complications, as well as an increased risk of premature illnesses and death later in life; all these issues are clear public-health concerns [2]. The rapid rise in childhood overweight emphasizes the importance of environmental factors on this issue [2–4]. Family influences - particularly parental influences - are the primary environmental components that researchers have evaluated in relation to the weight of children. Parents are the key agents of behaviour changes in their families as well as role models for their children’s physical appearance and health [5]. Although a long-term study suggests that parents might not be as successful as their children in weight maintenance, parents who participate in family-based pediatric weight controldo lose weight [6]. A correlation between the obesity of children and that of their parents exists [7, 8]. Numerous studies have shown that parental obesity can increase the risk of a child becoming obese [7–10].

Parental weight change is related to child weight change, according to family-based behavioral treatment programs for overweight and obese children [11–15]. Parental BMI change is a significant predictor of child weight; a reduction of one BMI unit in the parent is associated with a 0.255 reduction in her child’s BMI [11]. Parent and child weight change correlations have been uniformly positive during treatments, with correlations ranging from 0.31 to 0.76 [16–18]. Correlations over extended intervals have varied among the studies, with some studies [18] showing an improvement in the relationship over time, while others [16, 17] have shown a decrease. Parent weight is related to child weight, but it might not make an independent contribution. Instead, parent weight might be related to child weight through some of the same variables that predict child weight change, such as the child’s age and sex, the parental Body Mass Index (BMI), or family socioeconomic status (SES). Although insightful, these studies were limited in that they only predicted child BMI and child BMI-Z scores from a parent’s BMI [11, 15]. This present study provides the new or innovative ideas and design compared to the previous studies by involving fathers or mothers who gained weight or lost weight over time and investigating the association of the weight status change in the parent with that of the child during each follow-up period. The unique approach of our model by using parental weight changes as key predictors of child weight changes may be easily integrated in educational as well as health care systems.

Our study seeks to add to the above literature by evaluating the influence of parents who gained or lost weight on child’s weight via a 14-year examination of parent and child patterns. We use changes from the baseline as a key indicator to evaluate if these parental weight changes add significant incremental predictions when accounting for other factors.

## Methods

### Data sources

We employed a secondary analysis on the Indonesia Family Life Survey (IFLS), an ongoing national prospective longitudinal cohort study in Indonesia. The survey’s samples were collected between 1993 and 2007. The dataset is publicly available at RAND’s website (http://www.rand.org/labor/FLS/IFLS.html). We were granted access to the dataset by registering for access to the IFLS data download link. The survey sample represented about 83 % of the Indonesian population living in 13 of the country’s 27 major provinces [19]. The IFLS randomly selected 321 enumeration areas (EAs) within each of the 13 provinces chosen from a nationally representative sample frame used in the 1993 SUSENAS, a socioeconomic survey. The SUSENAS frame, designed by the Indonesian Central Bureau of Statistics (BPS), was based on the 1990 census. A complete interview was obtained for 7,039 households of the 7,730 households sampled (91.1 percent). Two children, aged 0 to 14, were randomly selected in each household. The eligible children included all biological, step, or adopted children of the household head and spouse, as well as any children fostered to any adult in the household.

### Ethical oversight

The survey and its procedures were properly reviewed and approved by IRBs in the USA (at RAND), in Indonesia, at Gajah Mada University (UGM), and earlier at the University of Indonesia (UI).

### Measurement

The anthropometric measurements (height and weight) and information regarding parental education, maternal employment, household income, and residence were collected from children under five years old and their parents in 1993. The same individuals were followed up at different points of time in 1997 (4–8 years old), 2000 (7–11 years old), and 2007 (14–18 years old). Specially trained members of the IFLS field team measured each individual’s height and weight without outdoor clothes or shoes, as per accepted international standards [20]. Standing height measures (for adults and children over age two) and recumbent length (for younger children) were taken using Shorr measuring boards; measures of weight were taken using Seca Model 770 scales, calibrated daily. Both of these measuring instruments have been used in survey work in other countries and are suitable for field work given their portability, durability, and accuracy. Children who were too young or not able to stand on their own were held by a parent and weighted (after the scale had been adjusted to zero with just the parent alone on the scale).

Height and weight were used to calculate Body Mass Index (BMI). BMI z-scores were determined for each child based on the 2006 WHO Child Growth Standards for children under five years old and the WHO Reference 2007 for children and adolescents, 5–18 years, age and gender specific. Underweight was defined as BMI z-score ≤ −2 SD for children aged 0–18 years. Normal weight was defined as −2 SD < BMI z-score < 2 SD (under 5 years) and −2 SD < BMI z-score < 1 SD (ages 5 to 18 years). Overweight was defined as 2 SD ≤ BMI z-score < 3 SD (under 5 years) and 1 SD ≤ BMI z-score < 2 SD (ages 5 to 18 years). Obese was defined as BMI z-score ≥ 3 SD (under 5 years) and BMI z-score ≥ 2 SD (ages 5 to 18 years) [21, 22]. Parent weight status according to BMI was determined using the WHO International Classification of adult underweight (BMI < 18.5 kg/m^2^), normal weight (18.5 kg/m^2^ ≤ BMI < 25.0 kg/m^2^), overweight (25.0 kg/m^2^ ≤ BMI < 30.0 kg/m^2^), and obesity (BMI ≥ 30.0 kg/m^2^) [23, 24].

There were 3,381 children under five years old in 1993. Of those, a total of 234 (6.9 %) children with missing data on height and weight or with only 1 height and 1 weight measurement during the 14 years of follow-up had to be eliminated from the sample. The final sample included 3,147 children. For further analysis to investigate the association of the weight status change in the parent with that of the child during each follow-up period, children and parents classified as underweight according to WHO at the baseline periods in 1993, 1997, and 2000 were also excluded, leaving normal weight, overweight, and obese status for the analysis. A child’s growth and development can be divided into four periods: preschool (under 5 years old), middle childhood (ages 4–8 years), school age (ages 7–11 years), and adolescence (ages 14–18 years). Parental weight gain was defined as an increase in a parent’s body weight during their children’s growth periods from 0–4 years to 4–8 years (first transition), from 4–8 years to 7–11 years (second transition), and from 7–11 years to 14–18 years (third transition). Parental weight loss was defined as a decrease in the parental body weight during each transition. We constructed two variables for parental weight change: (a) father’s weight change and (b) mother’s weight change. We classified each variable into two categories: positive change and negative change. A father’s or mother’s positive change was defined as the change in weight status in the father or mother who moved from an overweight status to a normal weight, from an obese status to a normal weight, or from an obese status to an overweight status during each transition. A father’s or mother’s negative change was defined as the change in weight status in a father or mother, when he or she moved from a normal weight to an overweight status, from a normal weight to an obese status, from an overweight status to an obese status, or when there was no change in an overweight or obese status during each transition. In the first, second, and third transition, the outcome variables were measured as continuous (child’s weight in kilograms in 1997, 2000, and 2007) and categorical (child’s weight change during each transition) variables.

### Potential covariates

Covariates included father’s education (none, elementary, junior high school, senior high school, and post-graduate), mother’s education (none, elementary, junior high school, senior high school, and post-graduate), maternal employment status (not working and working), parental BMI (both parents <25 kg/m^2^, only mother ≥25 kg/m^2^, only father ≥25 kg/m^2^, and both parents ≥25 kg/m^2^), household income (lowest, middle, highest), and residence (urban and rural). These covariates allowed for the control of variables that might influence child weight change.

### Statistical analyses

All statistical analyses were performed using SPSS 20.0 and SAS 9.3 for Windows. We used independent samples t-test to determine whether a difference in mean child weight existed based on parental weight gain or weight loss. Relative Risk (RR), controlled for covariates, estimated the ratio of the risk of having a child’s positive weight change with parents with positive weight changes against that with parents with negative weight changes. We set 0.05 as our alpha value for determining statistical significance.

## Results

**Fig. 1:**
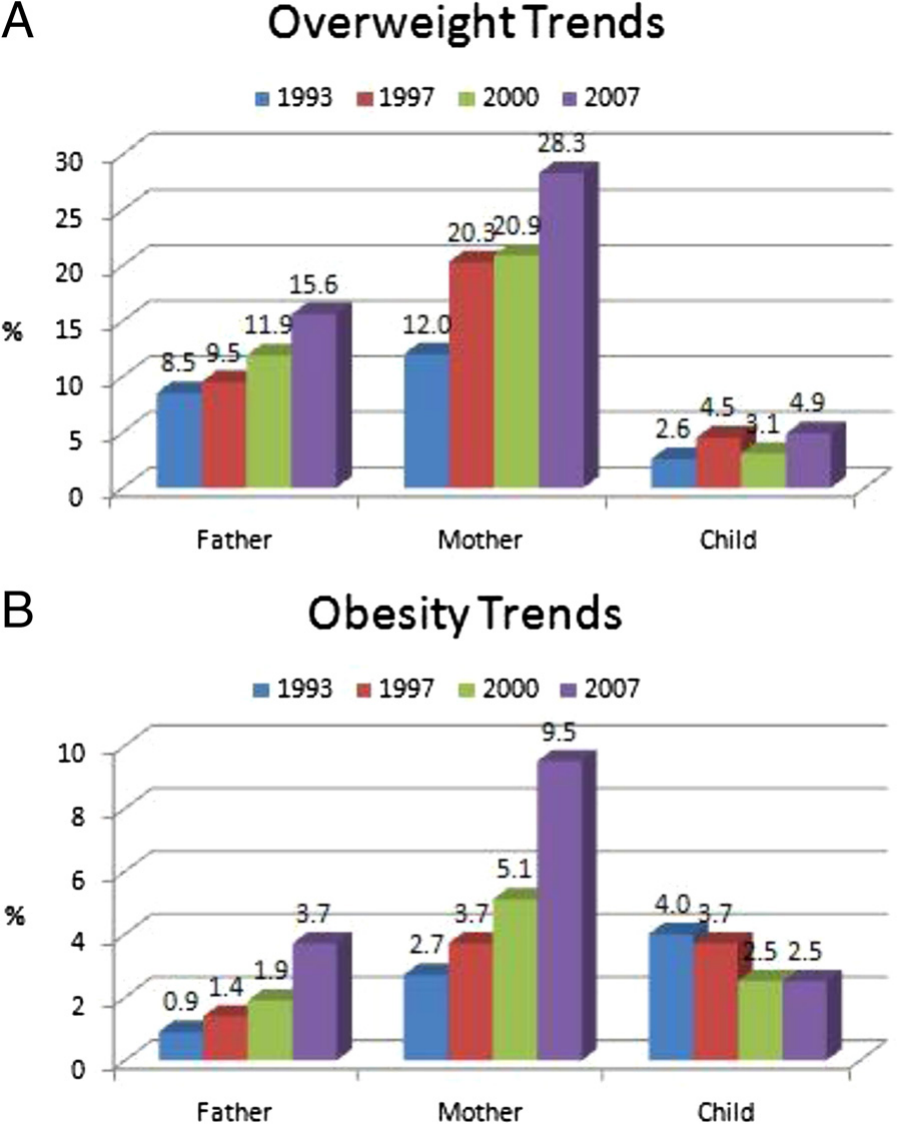
Prevalence of overweight (**a**) and obesity (**b**) among parents and children from 1993 to 2007

Figure 1 showed the trends in overweight and obesity prevalence between 1993 and 2007 for fathers, mothers, and children. The prevalence of overweight and obesity among fathers and mothers increased from 1993 to 2007. Among children, the prevalence of overweight increased from 1993 (2.6 %) to 1997 (4.5 %), decreased in 2000 (3.1 %), and increased again in 2007 (4.9 %). We found a decline in the obesity figures from 1993 (4.0 %) to 2007 (2.5 %).

A sample of children (1612 boys and 1535 girls) were aged 2.5 years (SD = 1.4) in 1993, had an average weight of 10.8 kg (SD = 3.3), aged 6.5 years (SD = 1.4) in 1997, had an average weight of 19.0 kg (SD = 3.1), aged 9.4 years (SD = 1.4) in 2000, had an average weight of 24.3 kg (SD = 4.3), and aged 16.4 years (SD = 1.4) in 2007, had an average weight of 51.1 kg (SD = 6.1). During the separate transitions, 1621 (61.2 %), 1630 (62.0 %), and 1611 (64.5 %) participating fathers and 1871 (70.3 %), 1610 (61.4 %), and 1779 (69.4 %) participating mothers gained weight, respectively. Table 1 showed that during each transition, parents who gained weight had a children with significantly higher weights than did parents who lost weight (p < 0.05). From the gender stratification, parents who gained weight had a higher child’s weight than did parents who lost weight, regardless of gender. However the association between father’s and child’s weight was statistically significant in girls only during the first and the second transitions and in boys only during the third transition. The association between mother’s and child’s weight was statistically significant in both boys and girls during the first and the second transitions, but statistically significant only for boys during the third transition.

**Table 1:** Means for child’s weight (kg) in 1997, 2000, and 2007 in relation to parental weight gain or weight loss during each transition, using independent samples t-test

0-4 years to 4–8 years	Child’s weight (kg)								
	Total			Boys			Girls		
	n	mean ± SE	*p*	n	mean ± SE	*p*	n	mean ± SE	*p*
Father			0.043			0.501	766		0.034
Weight gain	1621	19.24 ± 0.08		855	19.20 ± 0.11			19.28 ± 0.12	
Weight loss	1026	18.98 ± 0.10		496	19.08 ± 0.14		530	18.88 ± 0.15	
Mother			<0.001			<0.001			<0.001
Weight gain	1871	19.26 ± 0.08		954	19.28 ± 0.11		917	19.24 ± 0.12	
Weight loss	792	18.59 ± 0.09		411	18.70 ± 0.12		381	18.46 ± 0.12	
4-8 years to 7–11 years	n	mean ± SE	*p*	n	mean ± SE	*p*	n	mean ± SE	*p*
Father			0.005			0.434			0.002
Weight gain	1630	24.65 ± 0.11		823	24.32 ± 0.15		807	24.99 ± 0.16	
Weight loss	999	24.16 ± 0.14		526	24.13 ± 0.19		473	24.18 ± 0.22	
Mother			<0.001			<0.001			<0.001
Weight gain	1610	24.86 ± 0.11		830	24.74 ± 0.16		780	24.98 ± 0.16	
Weight loss	1013	23.68 ± 0.13		519	23.36 ± 0.15		494	24.02 ± 0.21	
7-11 years to 14–18 years	n	mean ± SE	*p*	n	mean ± SE	*p*	n	mean ± SE	*p*
Father			0.019			0.026			0.344
Weight gain	1611	51.24 ± 0.15		824	52.03 ± 0.23		787	50.41 ± 0.20	
Weight loss	882	50.64 ± 0.20		444	51.19 ± 0.30		438	50.09 ± 0.26	
Mother			0.008			0.002			0.564
Weight gain	1779	51.22 ± 0.15		895	52.11 ± 0.23		884	50.33 ± 0.19	
Weight loss	783	50.56 ± 0.20		414	50.96 ± 0.29		369	50.13 ± 0.27	

Father’s education, parental BMI, and residence were significantly associated with father’s weight changes. Table 2 displayed no significant decreased RR of the father’s positive weight change with his child’s positive weight change in the first (RR 0.64; 95 % CI: 0.35–1.17) and the third (RR 0.95; 95 % CI: 0.63–1.44) transitions. In the second transition, a father’s positive weight change led to an overall greater risk of having child’s positive weight change (RR 1.17; 95 % CI: 0.91–1.49), although the association was not significant. Multivariate analyses showed that a father’s positive weight change in the first (adjusted RR 0.74; 95 % CI: 0.42–1.31), second (adjusted RR 0.97; 95 % CI: 0.68–1.39), and third transitions (adjusted RR 0.98; 95 % CI: 0.62–1.56) remained not significant associated with the risk of a child’s positive weight change. Mother’s education, parental BMI, and residence were significantly associated with mother’s weight change. We found a mother’s positive weight change from the first (RR 1.74; 95 % CI: 1.22–2.48) and the second (RR 1.36, 95 % CI: 1.18–1.57) transitions to be significantly associated with her child’s positive weight change. In the third transition, we found no association between a mother’s positive weight change and her child’s positive weight change (RR 1.09; 95 % CI: 0.72–1.64). This relationship persisted after a full adjustment.

**Table 2:** Parental weight change in relation to child weight change during each transition

0-4 years to 4–8 years	Child Weight Change		cRR	aRR (95 % CI)
	Positive Change^a^	Negative Change^b^		
	(N = 181) n (%)	(N = 303) n (%)		
Father Weight Change^c^			0.64	0.74 (0.42 – 1.31)
Positive Change	8 (29.6)	19 (70.4)		
Negative Change	87 (46.3)	101 (53.7)		
Mother Weight Change^d^			1.74*	1.57* (1.03 – 2.41)
Positive Change	25 (54.3)	21 (45.7)		
Negative Change	48 (31.2)	106 (68.8)		
	Positive Change^a^	Negative Change^b^	cRR	aRR (95 % CI)
4-8 years to 7-11 years	(N = 507) n (%)	(N = 162) n (%)		
Father Weight Change^c^			1.17	0.97 (0.68 – 1.39)
Positive Change	33 (67.3)	16 (32.7)		
Negative Change	71 (57.7)	52 (42.3)		
Mother Weight Change^d^			1.36*	1.19* (1.01 – 1.41)
Positive Change	66 (85.7)	11 (14.3)		
Negative Change	119 (63.0)	70 (37.0)		
	Positive Change^a^	Negative Change^b^	cRR	aRR (95 % CI)
7-11 years to 14–18 years	(N = 206) n (%)	(N = 390) n (%)		
Father Weight Change^c^			0.95	0.98 (0.62 – 1.56)
Positive Change	19 (38.0)	31 (62.0)		
Negative Change	55 (39.9)	83 (60.1)		
Mother Weight Change^d^			1.09	1.26 (0.77 – 2.07)
Positive Change	20 (35.7)	36 (64.3)		
Negative Change	60 (32.8)	123 (67.2)		

Note. cRR = Crude Relative Risk; aRR = Adjusted Relative Risk; CI = Confidence Interval

*p < 0.05

^a^From overweight to normal, from obese to overweight or normal

^b^From normal to overweight or obese, from overweight to obese, from overweight to overweight, from obese to obese

^c^Adjusted for father’s education, parental BMI, and residence

^d^Adjusted for mother’s education, parental BMI, and residence

## Discussion

Our study was unique in its focus on the differences in a child’s weight during a parental weight gain or loss. It specifically investigated the weight change of both parents in relation to their child’s weight change. A father’s weight gain or weight loss was correlated to the weight of school-aged daughters but not associated with the weight of school-aged sons. A mother’s weight gain or weight loss was correlated to the weight of school-aged sons and school-aged daughters. A mother’s positive weight change also increased the chance of a positive weight change in pre-schoolers and school-aged children. As mothers tend to be the primary caregivers, they are perhaps a child’s role models, predominantly responsible for feeding their children, and monitoring the food intake of their children. They also likely eat with their children more frequently and are more dissatisfied with their bodies than fathers [25–28]. Mothers might be more likely to use feeding-based strategies that have been suggested to teach children emotional and disinhibited eating. They might also be more likely than fathers to employ authoritative and encourage compliance in their children [29, 30]. Most researchers have assumed that fathers do not have as much influence on a child’s development as do mothers [31]. However our study unearthed something new: parental weight gain or weight loss did not seem to be related to the weight of adolescent daughters. We found no associations between a parent’s positive weight change and the child’s positive weight change for children ranging from the age groups of “school-aged” to “adolescence” in the third transition. Our explanation is that the cognitive, physical, social, and lifestyle changes during adolescence can create profound changes in a child’s eating patterns. Teens tend to snack, miss meals, eat away from home, consume fast food, and start diets (especially true for females) more frequently than younger children [32]. Adolescents gave several reasons for not having family meals, including schedules differences, the desire for autonomy, and dissatisfaction with family relations. The most frequently mentioned reasons among parents were conflicting schedules due to work and activities such as sports [33].

The results of this study showed that parent weight change is a significant predictor and a key contributor to child weight change, a conclusion consistent with other research [11–17, 34]. Parental changes in eating and activity can act as models for changes in child behavior. A mother’s positive weight change increased the chance of a positive weight change in pre-schoolers and school-aged children but not in adolescents. However, during each transition, we found no associations between the father’s positive weight change and the child’s positive weight change, suggesting that the mechanisms affecting the child’s acquisition and maintenance of eating and exercise behaviors differ for each parent. Further studies might identify the exact mechanisms determining differences in the positive and negative weight changes in father-child and in mother-child pairings.

Whereas a parent’s self-control might weaken over time, child behavior can be maintained by consistent parental support or through child self-regulation [17]. Garn and Clark [7] suggested that childhood eating and exercise patterns are modeled after parental behaviors. Experimental research has indeed suggested that parental modeling can influence and alter children’s eating and exercise behavior [35–37]. Bandura [38] has argued that reciprocally reinforcing relationships among family members is important for acquiring and maintaining new behaviors. The family provides an ideal environment for the improvement of health-related behaviors, including eating and physical activity. Parental weight change might influence child weight change through the modification of the shared family environment, thereby facilitating weight changes in parents and their children.

Highly educated parents overweight or obese parents (BMI ≥25 kg/m^2^), and parents living in urban areas were more likely to have positive weight change. Having a post-graduate degree may result in better knowledge and understanding of healthy weight and weight control practices, including a healthy diet and adequate exercise. In addition, parents with higher education may increase access to healthy foods and the ability to maintain physical fitness [39]. Parental overweight or obesity probably contributes both genetic and family environmental influences for childhood overweight [10, 40]. Overweight parents tend to overcontrol the child’s feeding behaviours, food preferences and energy intake, such as restricting the total amount of food and pressuring children to eat healthy foods, possibly because they were more aware of acknowledging the problem and health consequences of obesity. These facts could perhaps be used to help their children, who are already overweight or obese, lead healthy lives. Parents’ restrictive eating behavior affects child’s weight, which in turn imposes positive weight change [41]. Though it has been estimated that overweight or obesity was observed to be more widespread in urban areas [42, 43], their awareness of the health threats that obesity poses is also increasing. A previous study shows that children in urban schools had more exercise equipment available at home, and were more frequently transported to places where they could be physically active [44]. In addition, a more competitive workplace environment in urban area and the insecurities associated with being discriminated and prejudiced both in their personal and professional lives often motivates urban adults who are overweight to make an effort to lose weight [45, 46].Our study has its strengths and weaknesses. This study adds to a small body of literature that supports the incremental effects of parental weight change on child weight change. Our dataset uses a rigorous sampling design that selects a large sample of a representative group of children and their parents among multiple time periods. The number of participants included in this study was larger than those of other studies. Additionally, this study includes repeated measures of a moderately sized cohort of parents and their children. Data from the same individuals over multiple points in time provides uniquely rich longitudinal data. The consecutive study (14-years) allowed us to identify developmental sequences for stability and continuity over time, thereby benefitting the quality of our assessment of the temporal generality of important key predictors of child weight change.

However, this study was limited by its use of BMI as a measure of weight status, a fact that might introduce misclassification problems and result in an estimation bias for the effects related to the relationship between parental and child changes. Another limitation is the likely existence other unmeasured parenting variables that contribute to child weight change, such as changes in the mood surrounding and discussions about eating and physical activity in the home. The impact of variables such as these deserves further research.

## Conclusions

Parental weight change is an independent predictor of child weight change. Positive weight change in the mother had a more dominant influence on a child’s positive weight change than did the father’s positive weight change. Understanding the relationships between parental weight change and child weight change during different transitions can help professionals educate parents about effective behavioral programs to use with their children. Future family-based obesity prevention and treatment programs should consider how best to include and engage mothers in their efforts to reduce obesity-related risk factors in the long term.
